# Protein composition of the occlusion bodies of *Epinotia aporema* granulovirus

**DOI:** 10.1371/journal.pone.0207735

**Published:** 2019-02-12

**Authors:** Tomás Masson, María Laura Fabre, María Leticia Ferrelli, Matías Luis Pidre, Víctor Romanowski

**Affiliations:** Instituto de Biotecnología y Biología Molecular (IBBM, UNLP-CONICET), Facultad de Ciencias Exactas, Universidad Nacional de La Plata, La Plata, Buenos Aires, Argentina; University of Rochester School of Medicine and Dentistry, UNITED STATES

## Abstract

Within family *Baculoviridae*, members of the *Betabaculovirus* genus are employed as biocontrol agents against lepidopteran pests, either alone or in combination with selected members of the *Alphabaculovirus* genus. *Epinotia aporema* granulovirus (EpapGV) is a fast killing betabaculovirus that infects the bean shoot borer (*E*. *aporema*) and is a promising biopesticide. Because occlusion bodies (OBs) play a key role in baculovirus horizontal transmission, we investigated the composition of EpapGV OBs. Using mass spectrometry-based proteomics we could identify 56 proteins that are included in the OBs during the final stages of larval infection. Our data provides experimental validation of several annotated hypothetical coding sequences. Proteogenomic mapping against genomic sequence detected a previously unannotated ac*110*-like core gene and a putative translation fusion product of ORFs *epap48* and *epap49*. Comparative studies of the proteomes available for the family *Baculoviridae* highlight the conservation of core gene products as parts of the occluded virion. Two proteins specific for betabaculoviruses (Epap48 and Epap95) are incorporated into OBs. Moreover, quantification based on emPAI values showed that Epap95 is one of the most abundant components of EpapGV OBs.

## Introduction

The family *Baculoviridae* comprises a diverse group of large double stranded DNA viruses that infect larvae of the insect orders *Lepidoptera*, *Hymenoptera* and *Diptera* [1]. Baculoviruses have a rod shaped, enveloped virion with a circular genome ranging from 80 to 180 kbp [2]. Virions are found in the environment embedded in a proteinaceous matrix that forms occlusion bodies (OBs), a phenotype that confers protection from adverse environmental conditions. OBs on leaves that are consumed by foraging larvae reach the midgut and, after being dissolved at high pH, release the occlusion derived viruses (ODVs), which initiate infection of the epithelial cells. These infected cells produce budded viruses (BVs) that disseminate the infection systemically [3]. Based on OBs morphology, baculoviruses were first classified in two groups: Nucleopolyhedrovirus (NPV) and Granulovirus (GV) [1]. Later, they were taxonomically divided into four genera: *Alphabaculovirus* (lepidopteran-specific NPV), *Betabaculovirus* (lepidopteran-specific GV), *Gammabaculovirus* (hymenopteran-specific NPV) and *Deltabaculovirus* (dipteran-specific NPV) [1].

Among different entomopathogenic viruses, the baculoviruses have received most of the attention due to their narrow host range which makes them safe pesticides. The majority of commercial products are based on virus isolates that belong to the genera *Alphabaculovirus* and *Betabaculovirus* [4]. The bean shoot borer (*Epinotia aporema*) is an oligophagous pest that attacks soybean crops [5]. A poliorganotropic fast killing betabaculovirus for this species, *Epinotia aporema* granulovirus (EpapGV), has been discovered and sequenced by our group [6, 7]. In order to improve our understanding of the infectious process we set out to analyze the protein content of EpapGV OB using a proteomic approach.

Mass spectrometry-based (MS) proteomics represents a powerful technique to interrogate the structural landscape of viral particles [8]. In addition to direct protein identification, spectral data derived from proteomic experiments can be used to identify novel features within genomic and transcriptomic datasets. This proteogenomic methodology is independent of reference annotation, thus providing excellent means for the refinement of gene models and the discovery of novel protein coding sequences [9].

Virion proteomics has been applied to study several DNA virus families (*Ascoviridae* [10], *Herpesviridae* [11], *Iridoviridae* [12], *Nudiviridae* [13] and *Poxviridae* [14]). In relation with the present study, eight ODV proteomes of baculoviruses have been analyzed (AcMNPV [15], AgMNPV [16], ChchNPV [17], HearNPV [18], MabrNPV [19], ClanGV [20], PiraGV [21] and CuniNPV [22]). These datasets point at a complex virion comprising a large number of proteins involved in virion morphogenesis, OBs formation and infection of insect midgut epithelial cells.

In this work we examined the protein content of EpapGV OBs using MS-based shotgun proteomics in order to describe the composition of this virion phenotype. A total of 56 viral proteins from EpapGV OBs were identified, the majority of which are conserved components among the members of the family *Baculoviridae* and few are betabaculovirus-specific proteins. Comparative proteomics of baculovirus showed a set of core gene products present in the majority of proteomes analyzed.

## Materials and methods

### Larvae and virus

Larvae of the bean shoot borer (*Epinotia aporema*), were collected from field in the experimental station of the Instituto Nacional de Tecnología Agropecuaria (INTA) and reared in our laboratory with an artificial diet and controlled light cycle (16 hours of light). The strain used in this study was EpapGV (Refseq ID NC_018875), collected in Oliveros (Santa Fe, Argentina) [6]. Permission was issued by INTA, which is the responsible authority for the experimental station where the collection of E. aporema larvae took place. At the time the virus was characterized, our group was collaborating with colleagues from INTA on this particular virus.

### Occlusion bodies (OBs) production and purification

Fourth instar *E*. *aporema* larvae were infected *per os* using artificial diet contaminated with a solution containing EpapGV OBs. Dying larvae with signs of infection were stored and processed as described previously [7]. Briefly, infected larvae were stored in distilled water and later homogenized in a Dounce homogenizer. The resulting solution was filtered through three layers of cheesecloth to eliminate insoluble insect debris. This extract was clarified by three steps of centrifugation at 10000 x g for 10 minutes followed by a wash with 0.05% v/v SDS solution. Clarified solution was subjected to ultracentrifugation in a continuous 30–60% w/w sucrose gradient (50000 x g, one hour, 4°C, Beckman SW 41 Ti rotor). The whitish/opalescent band corresponding to OBs was collected, diluted 10-fold in distilled water and pelleted by centrifugation at 14000 x g for 10 minutes. The final pellet was resuspended in distilled water and stored frozen at -20°C. Two biological independent samples were processed. Total protein mass in the sample was quantified using the Bradford assay [23].

### Mass spectrometry analysis

Protein digestion and analysis were performed at the Proteomics Core Facility CEQUIBIEM, at the University of Buenos Aires/CONICET (National Research Council) as follows: protein samples were reduced with 10 mM dithiothreitol in 50 mM ammonium bicarbonate pH 8 (45 min, 56°C) and carbamidomethylated with 20 mM iodoacetamide in the same solvent (40 min, room temperature, in darkness). This protein solution was precipitated with 0.2 volumes of 100% w/v trichloroacetic acid (Sigma) at -20°C for at least two hours and centrifuged at 12000 x g for 10 min (4°C). The pellet was washed twice with ice-cold acetone and dried at room temperature. Proteins were resuspended in 50 mM ammonium bicarbonate pH 8 and digested with trypsin (Promega V5111). The resulting peptides were desalted with ZipTip C18 columns (Millipore).

The digests were analyzed by nanoLC-MS/MS in a Thermo Scientific Q Exactive Mass Spectrometer coupled to a nanoHPLC EASY-nLC 1000 (Thermo Scientific). For the LC-MS/MS analysis, approximately 1 μg of peptides was loaded onto the column and eluted for 120 minutes using a reverse phase column (C18, 2 μm x 10 nm particle size, 50 μm x 150 mm) Easy-Spray Column PepMap RSLC (P/N ES801) suitable for separating complex mixtures of peptides with a high degree of resolution. The flow rate used for the nano-column was 300 nL min^-1^ and the solvent range from 7% B (5 min) to 35% B (120 min). Solvent A was 0.1% formic acid in water whereas B was 0.1% formic acid in acetonitrile. The injection volume was 2 μL. The MS equipment has a high collision dissociation cell (HCD) for fragmentation and an Orbitrap analyzer (Thermo Scientific, Q-Exactive). A voltage of 3.5 kV was used for Electro Spray Ionization (Thermo Scientific, EASY-SPRAY).

XCalibur 3.0.63 (Thermo Scientific) software was used for data acquisition and equipment configuration that allows peptide identification at the same time of their chromatographic separation. Full-scan mass spectra were acquired in the Orbitrap analyzer. The scanned mass range was 400–1800 m/z, at a resolution of 70000 at 400 m/z and the 12 most intense ions in each cycle, were sequentially isolated, fragmented by HCD and measured in the Orbitrap analyzer. Peptides with a charge of +1 or with unassigned charge state were excluded from fragmentation for MS2.

### Analysis of MS data

Q Exactive raw data was processed using Proteome Discoverer^TM^ software (version 2.1.1.21, Thermo Scientific) and searched against EpapGV protein database downloaded from NCBI (accession number NC_018875, National Center for Biotechnology Information; www.ncbi.nlm.nih.gov) digested with trypsin with a maximum of one missed cleavage per peptide. Proteome Discoverer^TM^ searches were performed with a precursor mass tolerance of 10 ppm and product ion tolerance of 0.05 Da. Static modifications were set to carbamidomethylation of Cys, and dynamic modifications were set to oxidation of Met and N-terminal acetylation. Protein hits were filtered for high confidence peptide matches with a maximum protein and peptide false discovery rate of 1% calculated using a reverse database strategy. The exponentially modified protein abundance index (emPAI) was calculated automatically by Proteome Discoverer^TM^ software and used to estimate the relative abundance of identified proteins within the sample.

### Non annotated peptides search

The complete genome sequence of EpapGV was translated *in silico* in all six frames using the Mascot search software. Spectral data was searched and all peptides hits were filtered to discard matches in previously annotated ORFs. The remaining peptides were mapped to the corresponding genomic position. Search for putative unannotated ORFs was done extending peptide hits until a stop codon was found at C-terminus, and a start or stop codon for the N-terminus. Homologous sequences were searched using the TBLASTN tool against all baculovirus genomes.

### Orthologs clustering

A database comprising all the ODV proteins detected in baculoviruses was generated using previous proteomic data sets [15–22]. The software BLASTP [24] and HHMER [25] were used to identify groups of orthologous proteins (orthogroups) among different proteomes by reciprocal best hits.

## Results

### Structural components of the EpapGV OB

**Fig 1:**
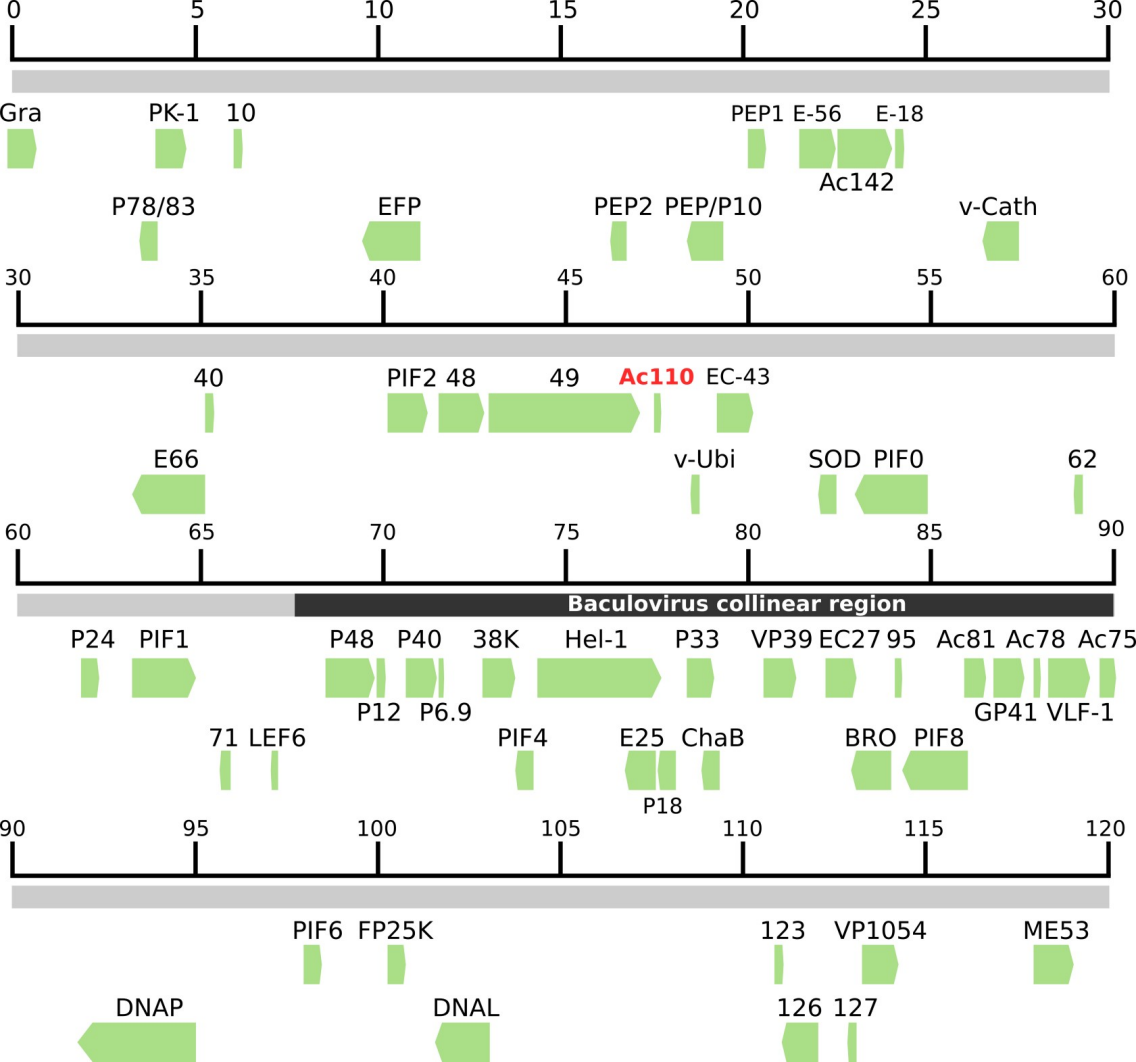
Genomic localization of ORFs coding for proteins found in EpapGV OBs. Proteins identified from spectral data (green arrows) were mapped to their respective genomic coordinate (grey line). The novel identified Ac110-like peptide is highlighted in red script. The baculovirus collinearity region is shown in black.

We determined the composition of purified EpapGV OBs employing a shotgun proteomic approach. The peptide mixture was separated by liquid chromatography and analyzed with tandem mass spectrometry (LC-MS/MS). This approach was used to avoid protein loss associated with SDS-PAGE gel extraction. We detected 56 proteins in our purified EpapGV OBs samples. Genes encoding these proteins comprise 43.93% of EpapGV total number of annotated ORFs (Fig 1), showing that a large part of the viral genome codes for structural proteins. A total of 10 proteins (Epap10, Epap62, Epap71, Epap123, LEF6, P6.9, Hel-1, P18, DNA Polymerase and DNA Ligase) were detected with only a single peptide, which might be related to low molar proportions of these polypeptides in the sample (Table 1). As additional evidence for the identification of these proteins, we checked the presence of several ion products belonging to the theoretical *b* and *y* spectral ions series for these peptides. The full list of proteins is shown in Table 1. In our samples we were unable to detect PIF3 and desmoplakin, two core gene products which have been confirmed in other virions by MS and western blot [15]. This could be attributed to proteolytic degradation, low protein level or deficient ionization of these proteins in our samples.

**Table 1:** Proteins detected in EpapGV OBs.

ORF	Protein	NCBI Protein Id.	Size (aa)	% Coverage	# Peptides	emPAI	% emPAI
1	Granulin	YP_006908509.1	248	60,48	25	1995261	857918
5	P78/83	YP_006908513.1	137	16,06	2	0,33	0,14
6	PK1	YP_006908514.1	276	30,43	9	3,44	1,48
10	Epap10	YP_006908518.1	90	23,33	1	0,33	0,14
14	EFP	YP_006908522.1	541	5,18	1	0,08	0,03
21	PEP2	YP_006908529.1	142	45,07	5	4,62	1,99
22	PEP/P10	YP_006908530.1	308	48,70	8	9	3,87
25	PEP1	YP_006908533.1	178	37,64	6	5,31	2,28
27	**PIF5**	YP_006908535.1	354	31,07	7	5,58	2,40
28	**Ac142**	YP_006908536.1	457	44,86	18	4,84	2,08
29	**ODV-E18**	YP_006908537.1	88	45,45	6	176,83	76,03
31	v-Cath	YP_006908539.1	329	5,77	2	0,29	0,12
39	ODV-E66	YP_006908547.1	654	33,64	14	5,31	2,28
40	Epap40	YP_006908548.1	102	75,49	6	16,78	7,22
47	**PIF2**	YP_006908555.1	374	20,05	7	0,69	0,30
48	Epap48	YP_006908556.1	446	37,67	13	2,54	1,09
49	Epap49	YP_006908557.1	1465	29,83	35	2,88	1,24
52	v-Ubi	YP_006908560.1	93	33,33	3	1,37	0,59
53	**ODV-EC43**	YP_006908561.1	348	47,41	12	6,20	2,67
58	SOD	YP_006908566.1	183	55,19	9	24,12	10,37
59	**PIF0**	YP_006908567.1	653	11,02	5	0,47	0,20
62	Epap62	YP_006908570.1	106	10,38	1	0,39	0,17
66	P24	YP_006908574.1	165	27,88	3	0,87	0,37
69	**PIF1**	YP_006908577.1	538	7,99	3	0,52	0,22
71	Epap71	YP_006908579.1	104	6,73	1	0,47	0,20
74	LEF6	YP_006908582.1	82	12,19	1	0,47	0,20
78	**P48/45**	YP_006908586.1	380	6,58	2	0,10	0,04
79	P12	YP_006908587.1	115	38,26	3	4,62	1,99
80	**P40**	YP_006908588.1	373	25,20	9	1,68	0,72
81	**P6.9**	YP_006908589.1	56	14,29	1	2,16	0,93
83	**38K**	YP_006908591.1	295	26,44	5	1,45	0,62
84	**PIF4**	YP_006908592.1	162	20,99	3	0,67	0,29
85	**Hel-1**	YP_006908593.1	1085	1,29	1	0	0,00
86	**ODV-E25**	YP_006908594.1	213	43,19	7	9	3,87
87	**P18**	YP_006908595.1	158	5,06	1	0	0.00
88	**P33**	YP_006908596.1	254	36,61	7	1,98	0,85
90	ChaB	YP_006908598.1	75	45,33	2	2,98	1,28
92	**VP39**	YP_006908600.1	293	75,43	21	232,27	100,00
93	**ODV-EC27**	YP_006908601.1	284	24,65	8	3,39	1,46
94	BRO	YP_006908602.1	359	5,85	2	0,23	0,10
95	Epap95	YP_006908603.1	73	69,86	5	176,83	76,03
96	**PIF8**	YP_006908604.1	567	14,10	7	1,22	0,52
98	**Ac81**	YP_006908606.1	191	23,04	5	1,61	0,69
99	**GP41**	YP_006908607.1	286	74,82	23	87,59	37,66
100	**Ac78**	YP_006908608.1	88	44,32	2	16,78	7,22
101	**VLF-1**	YP_006908609.1	368	35,87	11	3,06	1,32
103	Ac75	YP_006908611.1	149	56,38	9	7,11	3,06
106	**DNA Pol**	YP_006908614.1	1068	2,72	1	0	0.00
109	**PIF6**	YP_006908617.1	148	41,22	5	2,59	1,11
113	FP25K	YP_006908621.1	146	19,86	3	0,23	0,10
115	DNA Lig	YP_006908623.1	534	4,87	1	0,08	0,03
123	Epap123	YP_006908630.1	102	6,87	1	0,47	0,20
126	Epap126	YP_006908633.1	347	45,82	11	3,49	1,50
127	Epap127	YP_006908634.1	69	30,43	2	1,15	0,49
129	**VP1054**	YP_006908636.1	344	11,05	2	0,1	0,04
133	ME53	YP_006908640.1	373	9,91	2	0,22	0,09
-	**Ac110**	This study	47	29.79	1	-	-

% Coverage: percentage of the protein sequence covered by identified peptides.

emPAI: exponentially modified Protein Abundance Index.

ORF: Open Reading Frame, as numbered in EpapGV genome map published by Ferrelli *et al* [7].

# Peptides: number of individual peptides identified for each protein

Core gene products are in bold characters.

In addition to identifying the components of the OBs, we estimated the relative abundance of each protein; to this end we calculated the emPAI value proposed by Ishihama *et al* [26] for each protein. The emPAI value for the major capsid protein VP39 was used to normalize protein abundance (Table 1). Taking a cutoff value of at least 10% VP39 emPAI, the most abundant proteins are GP41, Granulin, ODV-E18, SOD and Epap95, together with VP39. The major capsid component VP39, the tegument protein GP41 and the major component of OB matrix granulin were expected to be among the most abundant proteins due to their known structural function. ODV-E18 ortholog in AcMNPV is an essential protein for BV production that also localizes to the ODV membrane [27]. Cu-Zn superoxide dismutase (SOD) activity in virion preparations of Chlorovirus PBCV-1 has been recently associated with reactive oxygen species reduction during the early stages of virus infection [28]. Finally, Epap95 a protein with orthologs in all the members of the genus *Betabaculovirus*, has been consistently detected in the granuloviruses of *Clostera anachoreta* (ClanGV) and *Pieris rapae* (PiraGV) as a component within ODVs [20–21].

Betabaculovirus gene content remains poorly characterized, with a large number of hypothetical genes predicted by phylogenomics methods. Some of these genes lack *bona fide* experimental evidence to confirm the actual existence of their putative protein products. Our proteomic data confirmed the presence of translation products for 10 hypothetical proteins annotated in the genome of EpapGV, namely, Epap10, Epap40, Epap48, Epap49, Epap62, Epap71, Epap95, Epap123, Epap126 and Epap127.

### Short peptides encoded in EpapGV genome that do not belong to annotated ORFs

To identify possible unannotated proteins, we searched our spectral data against a theoretical database comprising all translation products predicted in the six reading frames of EpapGV genome sequence (we included all possible ORFs, without introducing a minimal size criterion). We detected seven peptides that mapped to the EpapGV genome but did not belong to the set of annotated ORFs [7]. Their sequence and genomic location are detailed in S1 Appendix. One of these peptides matches an unannotated 47 amino acids long ORF overlapping *epap51* but in the opposite orientation. We further examined the presence of this novel ORF in other members of the family *Baculoviridae* and found that it is an ortholog of the core gene *ac110* [29]. This gene has been described as the *per os* infectivity factor 7 (*pif7*) and its product has only been detected in the proteome of HearNPV ODV [18] and EpapGV (this study). Genomic localization and orientation of this *ac110*-like gene is conserved within *Betabaculovirus*, providing additional evidence about its evolutionary conservation.

The remaining six peptides overlap with annotated ORFs (*chitinase*, *dna ligase* and *granulin*) or intergenic regions. Two peptides were found between ORFs *epap48* and *epap49* and one peptide between *epap61* and *epap62*. TBLASTN was used to find putative homologous unannotated peptides in other baculovirus genomes. Only the peptides overlapping with *chitinase* and *granulin* are conserved in homologous *loci* in GV and NPV genomes (S1 Appendix).

**Fig 2:**
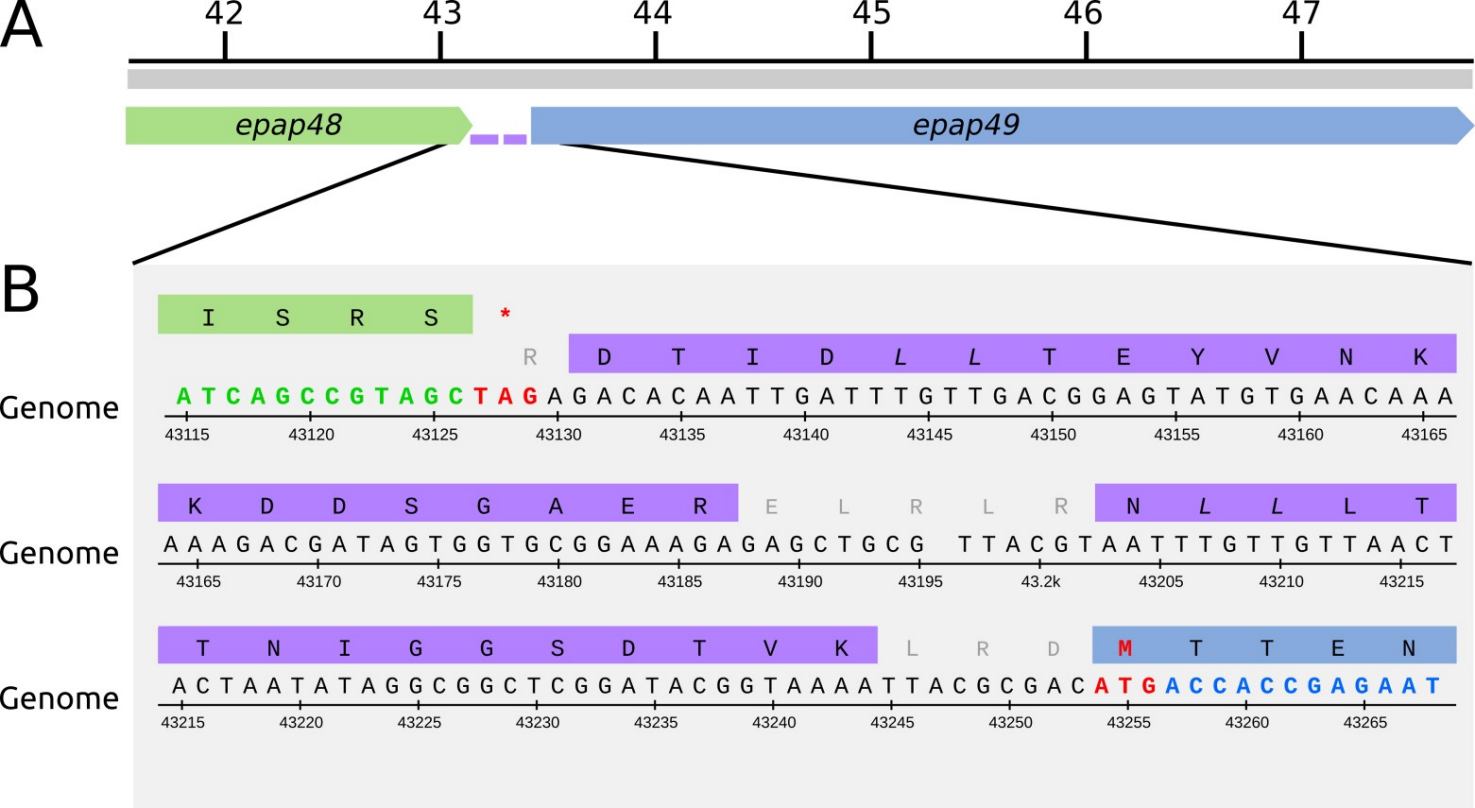
Putative fusion protein Epap48-Epap49. (A) Genomic locus containing *epap48* (green) and *epap49* (blue) genes. The two peptides detected by MS inside the intergenic region are depicted in purple. (B) Genome sequence and translation frame for each gene. Start and stop codons are shown in red (nucleotide numbers are those from NCBI accession number NC_018875).

Remarkably, peptides between *epap48* and *epap49* almost cover the entire 145 bp intergenic sequence (Fig 2). *Epap48* encodes a 446 amino acid long protein that is conserved in the betabaculoviruses. The putative translation product of *epap49* is a large protein composed of 1465 residues with no orthologs detected in other baculoviruses. Mapped peptides are located in the same reading frame as the translation product of *epap49*, but no methionine codon has been found in frame (Fig 2). One hypothesis that could explain the presence of these peptides is that Epap48 and Epap49 may be expressed as a fusion protein due to a putative +1 frameshifting event near the C-terminus of Epap48; further experimental validation of this potential fusion protein will be needed to confirm this hypothesis.

### Conservation of structural proteins in the family Baculoviridae

**Fig 3:**
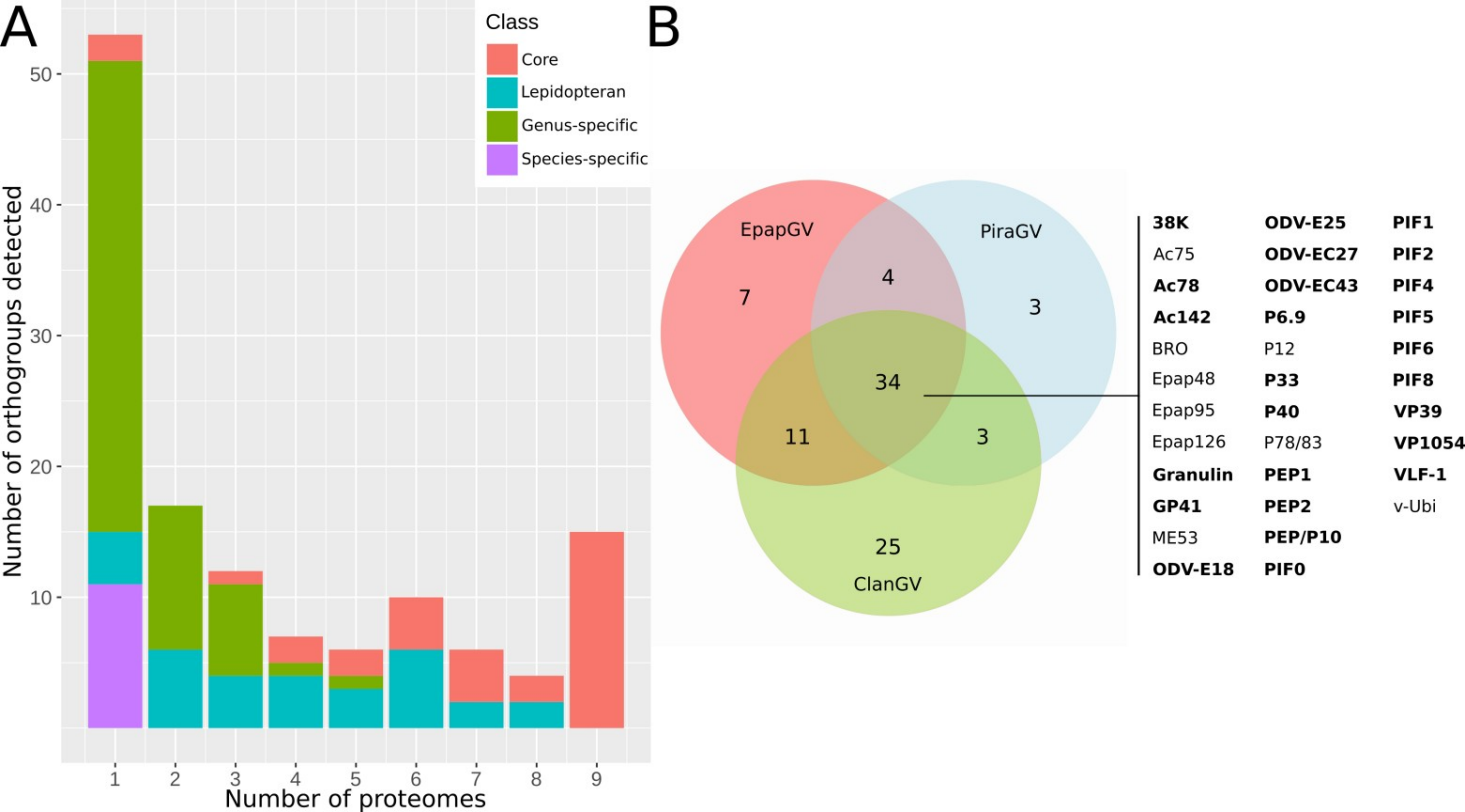
Baculovirus proteome comparison. (A) Protein conservation in baculovirus occluded virion proteomes. Proteins derived from baculoviral proteomics datasets were clustered in orthogroups. The conservations of each of these orthogroups was evaluated and scored according to the number of proteomes in which they were detected. Gene class distribution (core genes, lepidopteran baculovirus-specific, genus-specific, species-specific) of orthologs groups within *Baculoviridae* is highlighted in different colours. Hight of the bars reflects numbers of classes of proteins found in a given number of proteomes: *e*.*g*. the first bar (number of proteomes: 1) includes orthogroups present in just one of the reported nine baculoviral proteomes; the bar on the far right includes protein class counts (15 core genes) found in all the baculoviral OB proteomes. (B) Proteins detected in proteomes of betabaculoviruses were grouped in sets of orthologs and represented using a Venn diagram. A set of 34 proteins is present in all three viruses [20, 21, this study]; core gene products are highlighted in bold. Two of these protein clusters being specific of *Betabaculovirus* (Epap48 and Epap95).

The reports of OBs proteomes belonging to several baculoviruses were used to evaluate the conservation of the viral particle composition in this family. To date, eight proteomic studies were carried out on ODV, including five members of the genus *Alphabaculovirus* (AcMNPV, AgMNPV, ChchNPV, MabrNPV and HearNPV), two of the genus *Betabaculovirus* (ClanGV and PiraGV) and one of *Deltabaculovirus* (CuniNPV) [15–22]. Our study expands this data with the proteins present in the EpapGV OBs. Amino acid sequences of proteins detected in occluded virions of baculovirus were clustered in groups of orthologous proteins (orthogroups) using best reciprocal hit between proteomes (S1 Table). For each of these orthogroups we scored the number of proteomes in which they are present as a measure of their conservation. A class was assigned to each orthogroup based on their taxonomic distribution within the family *Baculoviridae* (core, lepidopteran-specific, genus-specific and species-specific) (Fig 3A). Most conserved protein groups (present in a larger number of proteomes) are enriched in core and lepidopteran-specific gene products. In contrast, proteins specific to a small set of proteomes are encoded by genus-specific and species-specific genes.

The betabaculovirus proteomes (EpapGV, ClanGV and PiraGV) were compared using a Venn diagram (Fig 3B). From the proteins present in all three viruses, BRO, Epap48, Epap95 and Epap126 are the only orthogroups without functional characterization. Interestingly, Epap95 is one of the most abundant proteins according to emPAI values. Additionally, Epap126 is shared between group II alphabaculoviruses and betabaculoviruses (except for ClanGV) (S1 Table). On the other hand, Epap10, Epap49 and Epap62 are proteins unique to EpapGV OBs. Remarkably, *epap10* orthologs are encoded only in five alphabaculoviruses that infect insects of the family *Tortricidae*, *Choristoneura fumiferana* NPV, *Choristoneura occidentalis* NPV, *Choristoneura rosaceana* NPV, *Cryptophlebia peltastica* NPV and *Epiphyas postvittana* NPV. This could be the product of an ancestral horizontal gene transfer between alphabaculoviruses and betabaculoviruses that coinfected the same host, based on gene conservation evidence.

## Discussion

**Fig 4:**
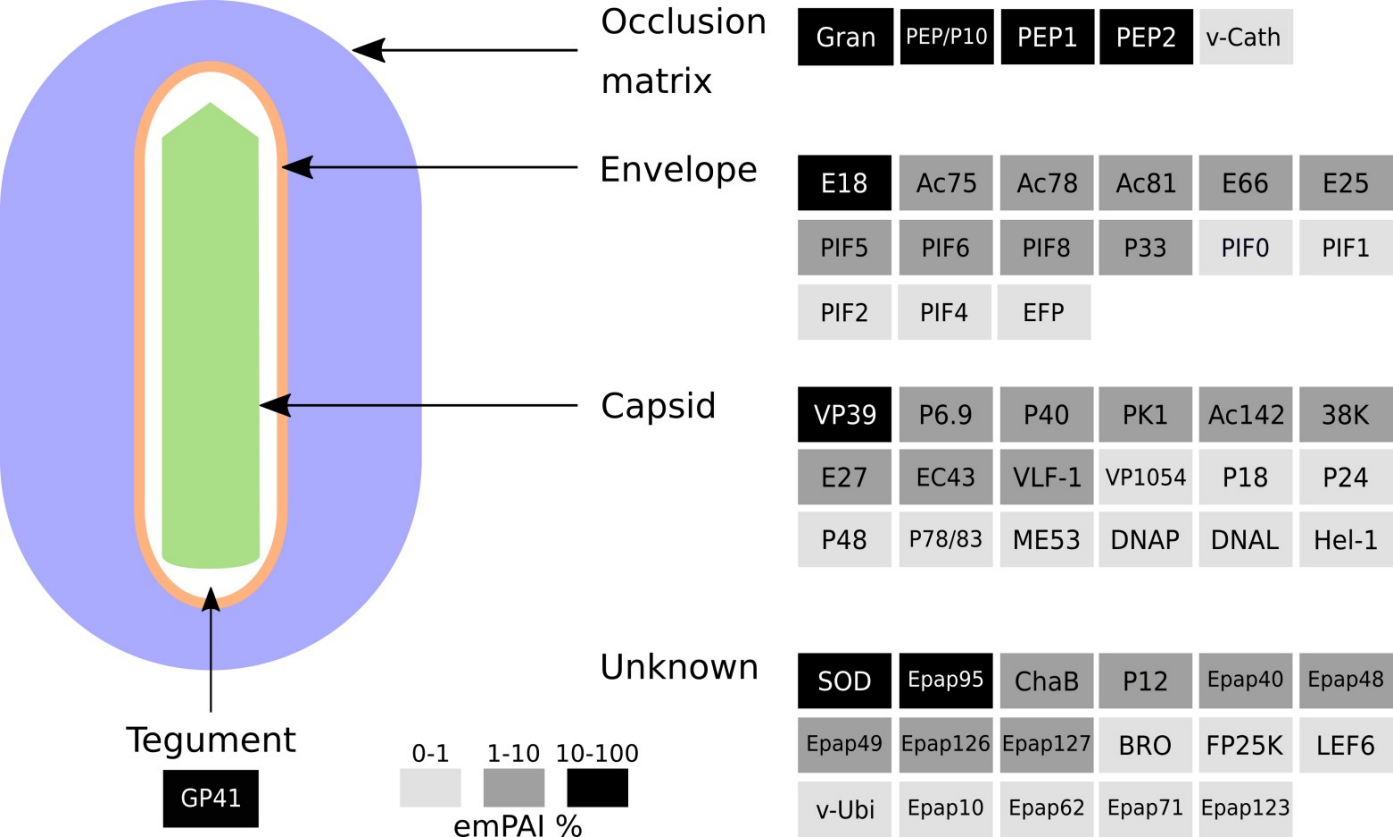
Schematic model of EpapGV OB. Qualitative and semi-quantitative virion model based on our proteomic data and localization described in published reports. Protein levels were estimated using the emPAI value and expressed as relative value with respect to the major capsid protein VP39.

Occluded virions are responsible for baculovirus primary infection. Proteome of OBs is related with oral infectivity, providing relevant information about conserved components potentially associated with midgut infection. Until now, the proteomes of ClanGV and PiraGV ODVs have been interrogated using MS-based techniques [20, 21]. These viral species are phylogenetically distant to EpapGV [7]. We explored possible divergence in protein composition employing a bottom-up proteomic approach to characterize the protein content of EpapGV OBs. A diagram of the EpapGV virion particle summarizing qualitative and semi-quantitative composition is shown in Fig 4. Virion components can be grouped in five classes based in their localization: 18 nucleocapsid proteins, 15 ODV envelope proteins, 5 occlusion matrix proteins, 1 tegument protein and 17 proteins of undefined localization. Comparisons across virion proteomes available for members of the family *Baculoviridae* highlighted the conservation of several structural components forming the mature virion. On the other hand, comparative genomics highlight the conservation of a collinear genomic region for lepidopteran-infecting baculoviruses [30]. Combined genomics and proteomics information suggests that this region, compared to the rest of genome, is densely populated by protein coding sequences corresponding predominantly to structural polypeptides (Fig 1 and S1 Fig). Intriguingly, the product of the core gene desmoplakin could not be detected in the betabaculovirus structural proteomes but it has been reported for alphabaculoviruses; we do not know if this is related to a different localization of this protein within betabaculovirus or due to technical reasons. In AcMNPV, desmoplakin has been implied in the segregation of nucleocapsids destined to build BV (which are ubiquitinated) and ODV (non ubiquitinated) [31].

It has been previously reported that proteins related to viral DNA metabolism and DNA binding capacity may be retained in the virion [15]. In the present study we could identify the DNA polymerase, DNA ligase and Helicase-1 in OBs. For other baculoviruses IE1, Alk-Exo, LEF1 and LEF3 have been detected also. This reinforces the idea that the viral DNA is associated with various proteins (in addition to the major condensing protein P6.9) inside the viral capsid.

The envelope that surrounds the ODV morphotype is especially adapted for primary infection of the insect midgut and presents a complex complement of proteins. These can be classified in two functional groups, those required for virion envelopment and those related with oral infectivity. Envelope morphogenesis begins with the formation of intranuclear microvesicles (IMV) derived from the inner nuclear membrane and the association with viral capsids. The ODV membrane proteins Ac75 and P18 are necessary for the generation of these IMV [32, 33]. Subsequently, envelopment of assembled nucleocapsids requires the ODV proteins Ac78, Ac81, Ac142, ODV-E25, ODV-EC43, P33 and P48 to form mature OBs [34–40]. On the other hand, several ODV membrane proteins are members of the PIF complex (PIF0, PIF1, PIF2, PIF3, PIF4, PIF5, PIF6 and PIF8); this molecular complex is the main effector of oral infection in the insect midgut. These proteins are encoded by core genes conserved in all the members of the *Baculoviridae* family [41].

The biological relevance of the betabaculovirus-specific orthogroups Epap48 and Epap95 within OBs is currently unknown. Moreover, the high content of Epap95 in the OBs may also be biologically relevant. On the other hand, Epap126 orthogroup is present in group II alphabaculoviruses and betabaculoviruses, which represents a conserved protein potentially involved in oral infection.

Baculovirus genomes are densely populated with coding sequences (overlapping in several cases) and contain short intergenic regions [2]. A recent study has described the transcriptional landscape of baculovirus infection, demonstrating the existence of several polycistronic and overlapping viral transcripts [42]. Together with other technologies, proteogenomic mapping is a valuable tool to improve the annotation of these complex coding regions. This approach has been used in proteome research for several virus families [43], but was applied only for one baculovirus, AgMNPV [44]. We identified seven peptides that do not map to previously annotated coding regions. One of these peptides turned to be an ortholog of *ac110*; this ORFs overlapped with the coding sequence of *epap51*. Moreover, the presence of peptides derived from alternative frames inside the coding regions of *granulin* and *chitinase* raises the question about the underlying complexity of baculovirus transcription and translation processes.

Surprisingly, two unmapped peptides were found to be encoded in the intergenic region of *epap48* and *epap49* and suggest the presence of a putative fusion product between these proteins. Epap49 is the largest protein annotated in EpapGV genome; it is 1465 amino acids long. As previously reported, it was difficult to annotate this as a hypothetical protein due to its atypically large size, absence of homologous proteins in Genbank and lack of known promoter motifs [7]. Also, it was noted that large proteins were coded in similar locations in the genomes of ChocGV and HearGV, 1144 and 1279 amino acids long, respectively [45, 46]. In the case of ChocGV it was not annotated in the genome [45] and in HearGV it was found to be a fusion of ORFs homologous to XecnGV 47 and 48 [46]. In this study, we found evidence that Epap49 is actually translated.

Data derived from comparative proteomics (Fig 3) highlight two prominent features of the baculoviral OB architecture. All baculoviral proteomes reported to date include 15 core gene products; additional core gene products were also found in fewer proteomes. Core and lepidopteran-specific gene products found in most of the proteomes might represent essential functions associated with these components (*e*.*g*. capsid translocation [31], *per os* infection [41] and structural integrity [36]). In contrast, several species-specific and genus-specific gene products are present in only one or a few number of proteomes, probably reflecting the result of the adaptation to a specific host.

## Conclusion

The protein composition of EpapGV OBs was interrogated using an MS-based proteomic approach. A total of 56 proteins have been detected in the EpapGV occluded virion, suggesting the presence of a highly conserved protein profile in baculoviral OBs. We identified Epap95, a betabaculovirs-specific protein, as a highly abundant capsid component. This protein represents an interesting candidate for further functional studies to explore its role in betabaculovirus pathogenesis. Through proteogenomic search we could detect a non-annotated coding region with a high degree of sequence identity to *ac110*. In addition, our data strongly suggest the translation of a putative fusion protein involving Epap48 and Epap49. Our study highlight the usefulness of MS proteomics to characterize the protein complement of the viral particle and the possibility to improve genome annotation through experimental evidence for translation of predicted coding regions.

## Supporting information

S1 Appendix EpapGV unannotated peptides detected by MS. (DOCX)

S1 Fig Parity plot of sequences coding for structural proteins present in AcMNPV, ChchNPV and PiraGV against EpapGV. (TIF)

S1 Table Proteomic profiles of baculovirus occluded virions proteomes. (DOCX)

## References

[1] Harrison RL, Herniou EA, Jehle JA, Theilmann DA, Burand JP, Becnel JJ, et al ICTV Virus Taxonomy Profile: Baculoviridae. Journal of General Virology. 2018;99(9):1185–1186. 10.1099/jgv.0.001107 . 29947603 PMC12662066

[2] van Oers MM, Vlak JM. Baculovirus genomics. Curr Drug Targets. 2007;8(10), 1051–68. 17979665 10.2174/138945007782151333

[3] Passarelli AL. Barriers to success: how baculoviruses establish efficient systemic infections. Virology. 2011;411(2):383–92. 10.1016/j.virol.2011.01.009 21300392 PMC3058624

[4] Haase S, Sciocco-Cap A, Romanowski V. Baculovirus insecticides in Latin America: historical overview, current status and future perspectives. Viruses. 2015;7:2230–67. 10.3390/v7052230 25941826 PMC4452904

[5] Sánchez NE, Pereyra PC. Neotropical soybean budborer, Crocidosema aporema (Walsingham, 1914) (Lepidoptera: Tortricidae) In Capinera J. L. (Ed.), Encyclopedia of Entomology. Dordrecht: Springer Netherlands 2008 pp. 2587–2588. 10.1007/978-1-4020-6359-6_2186

[6] Sciocco-Cap A, Parola AD, Goldberg AV, Ghiringhelli PD, Romanowski V. Characterization of a granulovirus isolated from Epinotia aporema Wals. (Lepidoptera: Tortricidae) larvae. Appl Environ Microbiol. 2001;67(8):3702–6. 10.1128/AEM.67.8.3702-3706.2001 11472950 PMC93074

[7] Ferrelli ML, Salvador R, Biedma M, Berretta M, Haase S, Sciocco-Cap A, et al Genome of Epinotia aporema granulovirus (EpapGV), a polyorganotropic fast killing betabaculovirus with a novel thymidylate kinase gene. BMC Genomics. 2012;13(1):548 10.1186/1471-2164-13-548 23051685 PMC3496565

[8] Greco TM, Diner BA, Cristea IM. The impact of mass spectrometry–based proteomics on fundamental discoveries in virology. Annu Rev Virol. 2014;1(1):581–604. 10.1146/annurev-virology-031413-085527 26958735 PMC6889812

[9] Nesvizhskii AI. Proteogenomics: concepts, applications and computational strategies. Nat Methods. 2014;11(11):1114–1125. 10.1038/nmeth.3144 25357241 PMC4392723

[10] Tan Y, Bideshi DK, Johnson JJ, Bigot Y, Federici BA. Proteomic analysis of the Spodoptera frugiperda ascovirus 1a virion reveals 21 proteins. J Gen Virol. 2009;90:359–365. 10.1099/vir.0.005934-0 19141444

[11] Vidick S, Leroy B, Palmeira L, Machiels B, Mast J, François S, et al Proteomic characterization of murid herpesvirus 4 extracellular virions. PLoS ONE. 2013;8(12):e83842 10.1371/journal.pone.0083842 24386290 PMC3875534

[12] Ince IA, Boeren SA, Van Oers MM, Vervoort JJM, Vlak JM. Proteomic analysis of Chilo iridescent virus. Virology. 2010;405(1):253–258. 10.1016/j.virol.2010.05.038 20598335 PMC7111926

[13] Bézier A, Harichaux G, Musset K, Labas V, Herniou EA. Qualitative proteomic analysis of Tipula oleracea nudivirus occlusion bodies. J Gen Virol. 2017;98(2):284–295. 10.1099/jgv.0.000661 28284235

[14] Doellinger J, Schaade L, Nitsche A. Comparison of the Cowpox Virus and Vaccinia Virus mature virion proteome: analysis of the species- and strain-specific proteome. PLOS ONE. 2015;10(11):e0141527 10.1371/journal.pone.0141527 26556597 PMC4640714

[15] Braunagel SC, Russell WK, Rosas-Acosta G, Russell DH, Summers MD. Determination of the protein composition of the occlusion-derived virus of Autographa californica nucleopolyhedrovirus. Proc Natl Acad Sci USA. 2003;100(17):9797–802. 10.1073/pnas.1733972100 12904572 PMC187845

[16] Braconi CT, Ardisson-Araújo DM, Paes Leme AF, Oliveira JV, Pauletti BA, Garcia-Maruniak A, et al Proteomic analyses of baculovirus Anticarsia gemmatalis multiple nucleopolyhedrovirus budded and occluded virus. J Gen Virol. 2014;95:980–989. 10.1099/vir.0.061127-0 24443474

[17] Xu F, Ince IA, Boeren S, Vlak JM, Van Oers MM. Protein composition of the occlusion derived virus of Chrysodeixis chalcites nucleopolyhedrovirus. Virus Res. 2011;158(1–2):1–7. 10.1016/j.virusres.2011.02.014 21354223

[18] Hou D, Zhang L, Deng F, Fang W, Wang R, Liu X, et al Comparative proteomics reveal fundamental structural and functional differences between the two progeny phenotypes of a baculovirus. J Virol. 2013;87(2):829–39. 10.1128/JVI.02329-12 23115289 PMC3554090

[19] Hou D, Chen X, Zhang LK. Proteomic analysis of Mamestra Brassicae Nucleopolyhedrovirus progeny virions from two different hosts. PLOS ONE. 2016; 11(4):e0153365 10.1371/journal.pone.0153365 27058368 PMC4825930

[20] Zhang X., Yin X., Liang Z., & Shao X. Proteomic analysis of the occlusion-derived virus of Clostera anachoreta granulovirus. J Gen Virol. 2015;96(8):2394–2404. 10.1099/vir.0.000146 25872743

[21] Wang XF, Zhang BQ, Xu HJ, Cui YJ, Xu YP, Zhang MJ, et al ODV-associated proteins of the Pieris rapae granulovirus. J Proteome Res. 2011;10(6):2817–2827. 10.1021/pr2000804 21517121

[22] Perera O, Green TB, Stevens SM Jr, White S, Becnel JJ. Proteins associated with Culex nigripalpus nucleopolyhedrovirus occluded virions. J Virol. 2007;81(9):4585–90. 10.1128/JVI.02391-06 17301145 PMC1900190

[23] Bradford MM. A rapid and sensitive method for the quantitation of microgram quantities of protein utilizing the principle of protein-dye binding. Anal. Biochem. 1976;72(1–2):248–54. 942051 10.1016/0003-2697(76)90527-3

[24] Altschul SF, Gish W, Miller W, Myers EW, Lipman DJ. Basic local alignment search tool. J. Mol. Biol. 1990;215(3):403–410. 10.1016/S0022-2836(05)80360-2 2231712

[25] Eddy SR. Accelerated Profile HMM Searches. PLoS Comput Biol. 2011;7(10):e1002195 10.1371/journal.pcbi.1002195 22039361 PMC3197634

[26] Ishihama Y, Oda Y, Tabata T, Sato T, Nagasu T, Rappsilber J, et al Exponentially modified protein abundance index (emPAI) for estimation of absolute protein amount in proteomics by the number of sequenced peptides per protein. Mol Cell Proteomics. 2005;4(9):1265–1272. 10.1074/mcp.M500061-MCP200 15958392

[27] McCarthy CB, Theilmann DA. AcMNPV ac143 (odv-e18) is essential for mediating budded virus production and is the 30th baculovirus core gene. Virology. 2008;375(1):277–291. 10.1016/j.virol.2008.01.039 18328526

[28] Kang M, Duncan GA, Kuszynski C, Oyler G, Zheng J, Becker DF, et al Chlorovirus PBCV-1 encodes an active copper-zinc superoxide dismutase. J Virol. 2014;88(21):12541–12550. 10.1128/JVI.02031-14 25142578 PMC4248938

[29] Javed MA, Biswas S, Willis LG, Harris S, Pritchard C, van Oers MM, et al Autographa californica multiple nucleopolyhedrovirus AC83 is a per os infectivity factor (PIF) protein required for occlusion-derived virus (ODV) and budded virus nucleocapsid assembly as well as assembly of the PIF complex in ODV envelopes. J Virol. 2017;91(5):e02115–16. 10.1128/JVI.02115-16 28031365 PMC5309931

[30] Zhu Z, Yin F, Liu X, Hou D, Wang J, Zhang L, et al Genome sequence and analysis of Buzura suppressaria nucleopolyhedrovirus: a group II Alphabaculovirus. PloS One. 2014;9(1):e86450 10.1371/journal.pone.0086450 24475121 PMC3901692

[31] Biswas S, Willis LG, Fang M, Nie Y, Theilmann DA. Autographa californica nucleopolyhedrovirus AC141 (Exon0), a potential E3 ubiquitin ligase, interacts with viral ubiquitin and AC66 to facilitate nucleocapsid egress. J Virol. 2018;92(3):e01713–17 10.1128/JVI.01713-17 29142135 PMC5774878

[32] Shi A, Hu Z, Zuo Y, Wang Y, Wu W, Yuan M, et al Autographa californica nucleopolyhedrovirus ac75 is required for the nuclear egress of nucleocapsids and intranuclear microvesicle formation. Journal of Virology. 2017;92(4):e01509–17. 10.1128/JVI.01509-17 29212928 PMC5790941

[33] Yuan M, Huang Z, Wei D, Hu Z, Yang K, Pang Y. Identification of Autographa californica nucleopolyhedrovirus ac93 as a core gene and its requirement for intranuclear microvesicle formation and nuclear egress of nucleocapsids. J Virol. 2011;85(22):11664–74. 10.1128/JVI.05275-11 21880748 PMC3209287

[34] Tao XY, Choi JY, Kim WJ, Lee JH, Liu Q, Kim SE, et al The Autographa californica multiple nucleopolyhedrovirus ORF78 is essential for budded virus production and general occlusion body formation. J Virol. 2013;87(15):8441–8450. 10.1128/JVI.01290-13 23698311 PMC3719795

[35] Dong F, Wang J, Deng R, Wang X. Autographa californica multiple nucleopolyhedrovirus gene ac81 is required for nucleocapsid envelopment. Virus Res. 2016;221:47–57. 10.1016/j.virusres.2016.05.005 27212683

[36] McCarthy CB, Dai X, Donly C, Theilmann DA. Autographa californica multiple nucleopolyhedrovirus ac142, a core gene that is essential for BV production and ODV envelopment. Virology. 2008;372(2):325–339. 10.1016/j.virol.2007.10.019 18045640

[37] Chen L, Hu X, Xiang X, Yu S, Yang R, Wu X. Autographa californica multiple nucleopolyhedrovirus odv-e25 (Ac94) is required for budded virus infectivity and occlusion-derived virus formation. Arch Virol. 2012;157(4):617–625. 10.1007/s00705-011-1211-9 22218963

[38] Alfonso V, Maroniche GA, Reca SR, López MG, del Vas M, Taboga O. AcMNPV Core gene ac109 is required for budded virion transport to the nucleus and for occlusion of viral Progeny. PLoS ONE. 2012;7(9):e46146 10.1371/journal.pone.0046146 23049963 PMC3458853

[39] Wu W, Passarelli AL. Autographa californica multiple nucleopolyhedrovirus Ac92 (ORF92, P33) is required for budded virus production and multiply enveloped occlusion-derived virus formation. J Virol. 2010;84(23):12351–61. 10.1128/JVI.01598-10 20861245 PMC2976406

[40] Yuan M, Wu W, Liu C, Wang Y, Hu Z, Yang K, et al A highly conserved baculovirus gene p48 (ac103) is essential for BV production and ODV envelopment. Virology. 2008;379(1):87–96. 10.1016/j.virol.2008.06.015 18656219

[41] Boogaard B, van Oers MM, van Lent JWM. An advanced view on baculovirus per os infectivity factors. Insects. 2018;9(3):E84 10.3390/insects9030084 30018247 PMC6164829

[42] Moldován N, Tombácz D, Szűcs A, Csabai Z, Balázs Z, Kis E, et al Third-generation sequencing reveals extensive polycistronism and transcriptional overlapping in a baculovirus. Sci. Rep. 2018;8(1):8604 10.1038/s41598-018-26955-8 29872099 PMC5988703

[43] Leroy B, Gillet L, Vanderplasschen A, Wattiez R. Structural proteomics of herpesviruses. Viruses. 2016;8(2):50 10.3390/v8020050 26907323 PMC4776205

[44] Brito AF, Braconi CT, Weidmann M, Dilcher M, Alves JMP, Gruber A, et al The Pangenome of the Anticarsia gemmatalis Multiple Nucleopolyhedrovirus (AgMNPV). Genome Biol Evol. 2015;8(1):94–108. 10.1093/gbe/evv231 26615220 PMC4758234

[45] Escasa SR, Lauzon HAM, Mathur AC, Krell PJ, Arif BM. Sequence analysis of the Choristoneura occidentalis granulovirus genome. J Gen Virol. 2006;87(7):1917–33. 10.1099/vir.0.81792-0 . 16760394

[46] Harrison RL, Popham HJR. Genomic sequence analysis of a granulovirus isolated from the Old World bollworm, Helicoverpa armigera. Virus Genes. 2008;36(3):565–81. 10.1007/s11262-008-0218-0 . 18418706

