# Supplementary material for: Protein composition of the occlusion bodies of Epinotia aporema granulovirus
Source: PLoS One. 2019 Feb 12;14(2):e0207735. doi: 10.1371/journal.pone.0207735 (PMC6372164; doi:10.1371/journal.pone.0207735)
Supplement: S1 Appendix — (DOCX) [file pone.0207735.s001.docx]

**S1 Appendix.** EpapGV unannotated peptides detected by MS

| Peptide Sequence | Length (aa) | Overlapping gene/between genes | Genome coordinates |
| --- | --- | --- | --- |
| **SWTCGSSSSLDR** | **12** | ***granulin* (same strand)** | **350 > 391** |
| **AVWIGGDGVV** | **10** | ***chitinase* (opposite strand)** | **28005 < 28037** |
| DTIDLLTEYVNKDDSGAER | 19 | Intergenic, *epap48* and *epap49* | 43128 > 43190 |
| NLLLTNIGGSDTVK | 14 | Intergenic, *epap48* and *epap49* | 43200 > 43247 |
| EQEIQYMLYEHR * | 12 | *epap51* (opposite strand) | 48549 > 48400 |
| ITIKFIK | 7 | Intergenic, *epap61* and *epap62* | 58524 > 58652 |
| SILILDGR | 8 | *dna ligase* (opposite strand) | 101551 > 101577 |

*Ac110, core gene product. Peptides with hits using TBLASTN are highlighted in bold.

For the peptides overlapping *granulin* and *chitinase* (shown in bold in the table) an extended translation product was obtained extending the sequence to both sides until finding stop codons. These extended peptides were used to search the database using TBLASTN in order to find homologous peptides in other baculoviruses. The extended peptides used were:

*granulin* overlapping peptide extended

SSCATGAEKSFFAKRGRVSFAKSFPSPPTKK**SWTCGSSSSLDR**CSPTAVTNLRCSTLSVLIPTMLPTTSSVSRILTMLAQTILSAST

TBLASTN hits (database:nr, max targ seq: 1000, word size 6, max evalue 0,0001):

135 hits: 48 GVs y 7 NPVs

*chitinase* overlapping peptide extended

MDRSESTHQSR**AVWIGGDGVV**

TBLASTN hits (database:nr restricted to baculoviridae and unclassified baculoviridae, max targ seq: 1000, word size: 3, max evalue: 10):

2 hits in 2 isolates of Plodia interpunctella GV
