# Supplementary figures and images for: Protein composition of the occlusion bodies of Epinotia aporema granulovirus
Source: PLoS One. 2019 Feb 12;14(2):e0207735. doi: 10.1371/journal.pone.0207735 (PMC6372164; doi:10.1371/journal.pone.0207735)

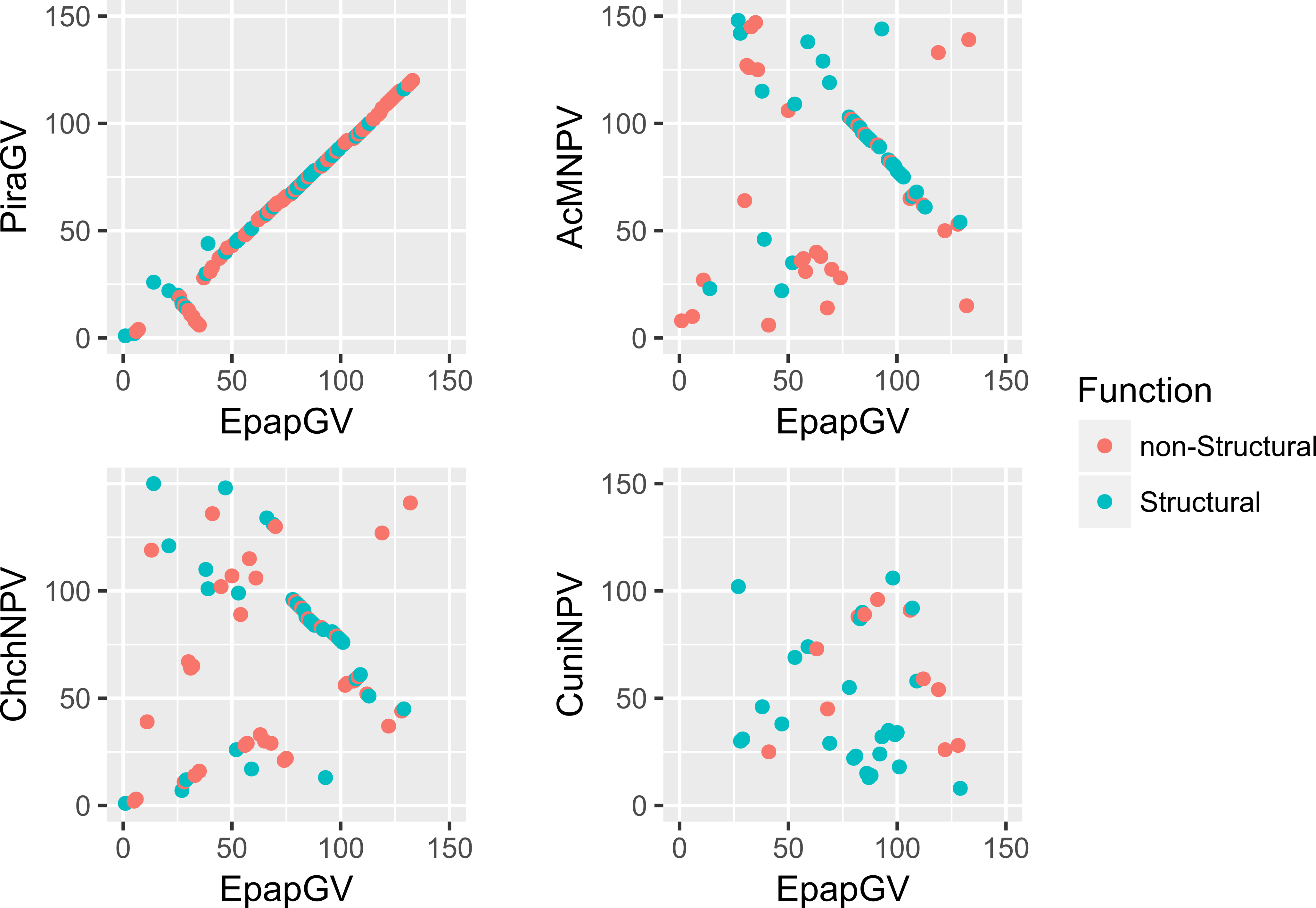

Supplement: S1 Fig — (TIF) [file pone.0207735.s002.tif]
