# Supplementary material for: Protein composition of the occlusion bodies of Epinotia aporema granulovirus
Source: PLoS One. 2019 Feb 12;14(2):e0207735. doi: 10.1371/journal.pone.0207735 (PMC6372164; doi:10.1371/journal.pone.0207735)
Supplement: S1 Table — (DOCX) [file pone.0207735.s003.docx]

**S1 Table.** Proteomic profiles of baculovirus occluded virions proteomes

|  | **Alphabaculovirus** | | | | | **Betabaculovirus** | | | **Deltabaculovirus** |
| --- | --- | --- | --- | --- | --- | --- | --- | --- | --- |
| **Orthogroup [#]** | **AcMNPV** | **AgMNPV** | **ChchNPV** | **HearNPV** | **MabrNPV** | **EpapGV** | **ClanGV** | **PiraGV** | **CuniNPV** |
| *Occlusion matrix* | | | | | | | | | |
| Polyhedrin [1] | 8 | 1 | 1 | 1 | 1 | 1 | 1 | 1 | - |
| PEP [2] | 131 | 31 | 121 | 120 | 53 | 21, 25 | 19, 36 | 20, 22 | - |
| P10 [3] | 137 | 24 | 18 | 21 | 152 | - | 16 | 17 | - |
| PEP/P10 [2, 3] | - | - | - | - | - | 22 | 35 | 21 | - |
| *ODV envelope* | | | | | | | | | |
| **ODV-E18** [4] | 143 | 18 | 12 | 10 | 159 | 29 | 13 | 14 | 31 |
| **ODV-E25** [5] | 94 | 71 | 86 | 82 | 87 | 86 | 76 | 76 | 15 |
| ODV-E66 [6] | 46 | 114 | 101 | 96 | 69, 136 | 39 | - | 39, 44 | - |
| **P33** [7] | 92 | 73 | 84 | 80 | 88 | 88 | 78 | 78 | 14 |
| **PIF0** [8] | 138 | 23 | 17 | 20 | 153 | 59 | 49 | 51 | 74 |
| **PIF1** [8] | 119 | 43 | 131 | 111 | 43 | 69 | 61 | 61 | 29 |
| **PIF2** [8] | 22 | 155 | 148 | 132 | 42 | 47 | 23 | 40 | 38 |
| **PIF3** [8] | 115 | 46 | 110 | 98 | 59 | 38 | 33 | 30 | 46 |
| **PIF4** [8] | 96 | 69 | 88 | 85 | 84 | 84 | 74 | 74 | 90 |
| **PIF5** [8] | 148 | 13 | 7 | 15 | 6 | 27 | 15 | 16 | 102 |
| **PIF6** [8] | 68 | 94 | 61 | 64 | 104 | 109 | 98 | 96 | 58 |
| **PIF8** [8] | 83 | 80 | 81 | 76 | 93 | 96 | 86 | 85 | 35 |
| AC75 [9] | 75 | 88 | 57 | 69 | 108 | 103 | 94 | 92 | - |
| **Ac78** [10] | 78 | 85 | 77 | 72 | 97 | 100 | 90 | 89 | 34 |
| **Ac81 [**11] | 81 | 82 | 79 | 74 | 95 | 98 | 88 | 87 | 106 |
| **Ac110** [12] | 110 | 51 | 98 | 93 | 73 | this study | 20 | 44 | 70 |
| **GP41** [13] | 80 | 83 | 78 | 73 | 96 | 99 | 89 | 88 | 33 |
| F-protein [14] | 23 | 143 | 150 | 133 | 8 | 14 | 30 | 26 | 104 |
| *Nucleocapsid* | | | | | | | | | |
| P78/83 [15] | 9 | 2 | 2 | 2 | 2 | 5 | 2 | 2 | - |
| **VP39** [16] | 89 | 76 | 82 | 78 | 91 | 92 | 81 | 81 | 24 |
| **p6.9** [17] | 100 | 66 | 93 | 88 | 78 | 81 | 71 | 71 | 23 |
| **P40** [18] | 101 | 65 | 94 | 89 | 77 | 80 | 70 | 70 | 22 |
| **P48/45** [19] | 103 | 63 | 96 | 89 | 75 | 78 | 68 | 68 | 55 |
| **Ac142** [20] | 142 | 19 | 11 | 9 | 160 | 28 | 14 | 15 | 30 |
| **ODV-EC43** [21] | 109 | 53 | 99 | 94 | 72 | 53 | 44 | 46 | 69 |
| P24 [22] | 129 | 33 | 134 | 118 | 11 | 66 | 57 | 58 | - |
| **ODV-EC27** [23] | 144 | 17 | 13 | 11 | 158 | 93 | 82 | 82 | 32 |
| **VLF-1** [24] | 77 | 86 | 76 | 71 | 98 | 101 | 91 | 90 | 18 |
| **38K** [25] | 98 | 68 | 91 | 86 | 80 | 83 | 73 | 73 | 87 |
| **VP1054** [26] | 54 | 107 | 45 | 47 | 125 | 129 | 119 | 116 | 8 |
| **P18** [27] | 93 | 72 | 85 | 81 | 86 | 87 | 77 | 77 | 13 |
| ME53 [28] | 139 | 21 | 8 | 16 | 7 | 133 | 123 | 120 | - |
| PK1 [29] | 10 | 3 | 3 | 3 | 3 | 6 | 3 | 3 | - |
| VP80 [30] | 105 | 62 | 97 | 92 | 74 | - | - | - | - |
| *Unknown localization* | | | | | | | | | |
| FP25K [31] | 61 | 101 | 51 | 50 | 116 | 113 | 102 | 100 | - |
| P12 [32] | 102 | 64 | 95 | 90 | 76 | 79 | 69 | 69 | - |
| **Desmop** [33] | 66 | 96 | 59 | 66 | 106 | 107 | 96 | 94 | 92 |
| v-Ubi [34] | 35 | 138 | 26 | 28 | 145 | 52 | 43 | 45 | - |
| SOD [35] | 31 | 131 | 115 | 106 | 57 | 58 | 48 | 50 | - |
| *DNA binding capacity* | | | | | | | | | |
| **LEF1** [36] | 14 | 148 | 138 | 124 | 29 | 68 | 60 | 60 | 45 |
| **DNA Pol** [37] | 65 | 97 | 58 | 67 | 107 | 106 | 95 | 93 | 91 |
| LEF3 [38] | 67 | 95 | 60 | 65 | 105 | 108 | 97 | 95 | - |
| **Hel-1** [39] | 95 | 70 | 87 | 84 | 85 | 85 | 75 | 75 | 89 |
| IE1 [40, 41] | 147 | 14 | 16 | 14 | 157 | 35 | 6 | 6 | - |
| LEF6 [42] | 28 | 124 | 21 | 24 | 149 | 74 | 65 | 65 | - |
| **Alk-Exo** [43] | 133 | 29 | 127 | 114 | 47 | 119-120 | 109 | 107 | 54 |
| *Minor components* | | | | | | | | | |
| Hear44 | - | - | 42 | 44 | 128 | 126 | 116 | 114 | - |
| Hear45 | - | - | 43 | 45 | 127 | 127 | 117 | 115 | - |
| HCF-1 | 70 | - | - | - | - | - | - | - | - |
| P43 | 39 | - | - | - | - | - | - | - | - |
| PCNA | 49 | - | 66 | - | - | - | - | - | - |
| PNK/PNL | 86 | 57 | - | - | - | - | - | - | - |
| Ac30 | 30 | 126 | - | - | - | - | - | - | - |
| Ac5 | 5 | 165 | - | - | - | - | - | - | - |
| Ac74 | 74 | 89 | - | - | - | - | - | - | - |
| Ac79 | 79 | 84 | - | - | 15 | - | - | - | - |
| ChaB1 | 58/59 | 103 | 49 | 51 | 121 | - | - | - | - |
| ODV-E26 | 16 | 150 | - | - | - | - | - | - | - |
| CG30 | 88 | 77 | - | 77 | 92 | - | - | - | - |
| Ac114 | 114 | 47 | - | - | - | - | - | - | - |
| Ac132 | 132 | 30 | - | - | - | - | - | - | - |
| PTP1/2 | 1 | 160 | 140 | - | 32 | - | - | - | - |
| Ac18 | 18 | 152 | 125 | - | 49 | - | - | - | - |
| EGT | 15 | 149 | 141 | 126 | 33 | 132 | 122 | 119 | - |
| 38.7 kDa | 13 | 147 | 137 | 123 | 30 | 67 | 59 | 59 | - |
| PP31 | 36 | 140 | 28 | 31 | 143 | - | - | - | - |
| IAP | several | 123 | 39 | several | 131 | several | 100 | several | - |
| CBP | 145 | 16 | 14 | 12 | 157 | 33 | 8 | 8 | - |
| Ac146 | 146 | 15 | 15 | 13 | 156 | 34 | 7 | 7 | - |
| Ac34 | 34 | 137 | 25 | 27 | 146 | - | - | - | - |
| BJDP | 51 | 110 | 38 | 39 | 132 | - | - | - | - |
| Ac48 | 48 | 113 | - | - | - | 123 | 113 | 111 | - |
| Chch46 | - | - | 46 | 48 | 124 | - | - | - | - |
| Ac56 | 56 | 105 | 47 | 49 | 123 | - | - | - | - |
| ChaB2 | 60 | 102 | 50 | 52 | 120 | 90 | - | - | - |
| Ac108 | 108 | 54 | 100 | 95 | 71 | - | - | - | - |
| Chch105 | - | - | 105 | - | - | - | - | - | - |
| PARG | - | - | 108 | 100 | 61 | - | - | - | - |
| Chch123 | - | - | 123 | - | 50 | - | - | - | - |
| GP16 | 130 | 32 | 133 | 119 | 10 | - | - | - | - |
| Chch135 | - | - | 135 | - | - | - | - | - | - |
| HOAR | - | - | 4 | 4 | 4 | - | - | - | - |
| LEF12 | 41 | 118 | - | 36 | - | - | - | - | - |
| **Ac53** | 53 | 109 | 41 | 43 | 129 | 125 | 115 | 113 | - |
| Ac76 | 76 | 87 | 56 | 70 | 109 | 102 | 92 | 91 | - |
| Hear83 | - | - | - | 83 | 37 | - | - | - | - |
| Ac63 | 63 | - | 31 | 121 | - | - | - | - | - |
| Mabr52 | 151 | - | - | - | 17, 52, 110 | - | - | - | - |
| Mabr21 | - | - | - | - | 21 | - | - | - | - |
| Mabr23 | - | - | - | - | 23 | - | - | - | - |
| Helicase-2 | - | - | - | - | 25 | 119-120 | - | 108 | - |
| v-Cath | 127 | - | 64 | 56 | 27 | 31 | 11 | 11 | - |
| Ac4 | 4 | 164 | - | - | 38, 64 | - | - | - | - |
| RR | - | - | 122, 151 | - | 51, 162 | - | - | - | - |
| Mabr56 | - | - | - | - | 56 | - | - | - | - |
| Mabr58 | - | - | 111 | - | 58 | - | - | - | - |
| NRK1 | 33 | 136 | 106 | - | 63 | 61 | - | - | - |
| VEF | - | - | - | - | 81 | - | - | - | - |
| Mabr83 | - | - | - | - | 83 | - | - | - | - |
| TLP-20 | 82 | 81 | 80 | 75 | 94 | 97 | 87 | 86 | - |
| Mabr100 | - | - | - | 34 | 100 | - | - | - | - |
| Mabr112 | - | - | - | - | 112 | - | - | - | - |
| Ac43 | 43 | 116 | 35 | 37 | 135 | - | - | - | - |
| Ac26 | 26 | 122 | 23 | 26 | 147 | - | - | - | - |
| DBP | 25 | 121 | 22 | 25 | 148 | 75 | 66 | 66 | - |
| P26 | 136 | 25 | 63 | 22 | 151 | - | - | - | - |
| *Betabaculovirus-specific* | | | | | | | | | |
| Epap10 | - | - | - | - | - | 10 | - | - | - |
| Epap40 | - | - | - | - | - | 40 | 39 | 31 | - |
| Epap48 | - | - | - | - | - | 48 | 22 | 42 | - |
| Epap49 | - | - | - | - | - | 49 | - | - | - |
| Epap62 | - | - | - | - | - | 62 | 54 | 55 | - |
| Epap71 | - | - | - | - | - | 71 | 63 | 63 | - |
| Epap95 | - | - | - | - | - | 95 | 84 | 84 | - |
| Pira29 | - | - | - | - | - | - | - | 29 | - |
| Pira54 | - | - | - | - | - | - | - | 54 | - |
| Clan17 | - | - | - | - | - | - | 17 | 18 | - |
| Clan18 | - | - | - | - | - | 26 | 18 | 19 | - |
| Clan20 | - | - | - | - | - | - | 20 | - | - |
| Clan27 | - | - | - | - | - | - | 27 | 35 | - |
| Clan32 | - | - | - | - | - | 37 | 32 | 28 | - |
| Clan37 | - | - | - | - | - | - | 37 | - | - |
| Clan42 | - | - | - | - | - | - | 42 | - | - |
| Clan45 | - | - | - | - | - | - | 45 | 47 | - |
| Clan79 | - | - | - | - | - | - | 79 | - | - |
| Clan93 | - | - | - | - | - | - | 93 | - | - |
| Clan08 | - | - | - | - | - | - | 108 | 106 | - |

# References to major proteins present in several baculoviruses

Number inside cells correspond to the ORF numbers according to reference sequence assembly available in NCBI (National Center for Biotechnology Information; www.ncbi.nlm.nih.gov).

Cells shaded represent proteins detected in MS experiments.

Orthogroups in bold denote core gene products.

**References**

1. Yang S, Zhao L, Ma R, Fang W, Hu J, Lei C, et al. Improving baculovirus infectivity by efficiently embedding enhancing factors into occlusion bodies. Appl. Environ. Microbiol. 2017;83(14) pmid:28500037.
2. Li J, Zhou Y, Lei C, Fang W, Sun X. Improvement in the UV resistance of baculoviruses by displaying nano-zinc oxide-binding peptides on the surfaces of their occlusion bodies. Appl. Microbiol. Biotechnol. 2015;99(16):6841–53. pmid: 25895092
3. Wang L, Salem TZ, Campbell DJ, Turney CM, Kumar CMS, Cheng XW. Characterization of a virion occlusion-defective Autographa californica multiple nucleopolyhedrovirus mutant lacking the p26, p10 and p74 genes. J Gen Virol. 2009;90(7):1641–8. pmid:19264658.
4. McCarthy CB, Theilmann DA. AcMNPV ac143 (odv-e18) is essential for mediating budded virus production and is the 30th baculovirus core gene. Virology. 2008;375(1):277–91. pmid:18328526.
5. Chen L, Hu X, Xiang X, Yu S, Yang R, Wu X. Autographa californica multiple nucleopolyhedrovirus odv-e25 (Ac94) is required for budded virus infectivity and occlusion-derived virus formation. Arch. Virol. 2012;157(4):617–25. pmid:22218963.
6. Xiang X, Chen L, Hu X, Yu S, Yang R, Wu X. Autographa californica multiple nucleopolyhedrovirus odv-e66 is an essential gene required for oral infectivity. Virus Res. 2011;158(1–2):72–8. pmid:21440017.
7. Wu W, Passarelli AL. Autographa californica multiple nucleopolyhedrovirus Ac92 (ORF92, P33) is required for budded virus production and multiply enveloped occlusion-derived virus formation. J. Virol. 2010;84(23):12351–61. pmid:20861245.
8. Boogaard B, van Oers MM, van Lent JWM. An Advanced View on Baculovirus per Os Infectivity Factors. Insects. 2018;9(3):E84 pmid: 30018247
9. Shi A, Hu Z, Zuo Y, Wang Y, Wu W, Yuan M, et al. Autographa californica Nucleopolyhedrovirus ac75 is Required for the Nuclear Egress of Nucleocapsids and Intranuclear Microvesicle Formation. J. Virol. 2017;92(4):JVI.01509-17. pmid:29212928.
10. Tao XY, Choi JY, Kim WJ, Lee JH, Liu Q, Kim SE, et al. The Autographa californica Multiple Nucleopolyhedrovirus ORF78 Is Essential for Budded Virus Production and General Occlusion Body Formation. J. Virol. 2013;87(15):8441–50. pmid:23698311.
11. Dong F, Wang J, Deng R, Wang X. Autographa californica multiple nucleopolyhedrovirus gene ac81 is required for nucleocapsid envelopment. Virus Res. 2016;221:47–57. pmid:27212683.
12. Javed MA, Biswas S, Willis LG, Harris S, Pritchard C, van Oers MM, et al. Autographa californica Multiple Nucleopolyhedrovirus AC83 is a Per Os Infectivity Factor (PIF) Protein Required for Occlusion-Derived Virus (ODV) and Budded Virus Nucleocapsid Assembly as well as Assembly of the PIF Complex in ODV Envelopes. J Virol. 2017;91(5):e02115-16. pmid: 28031365
13. Olszewski J, Miller LK. A Role for Baculovirus GP41 in Budded Virus Production. Virology. 1997;233(2):292–301. pmid:9217053.
14. Lung O, Westenberg M, Vlak JM, Zuidema D, Blissard GW. Pseudotyping Autographa californica multicapsid nucleopolyhedrovirus (AcMNPV): F proteins from group II NPVs are functionally analogous to AcMNPV GP64. J. Virol. 2002;76(11):5729–36. pmid:11992001.
15. Ohkawa T, Volkman LE, Welch MD. Actin-based motility drives baculovirus transit to the nucleus and cell surface. J. Cell Biol. 2010;190(2):187–95. pmid:20660627.
16. Katsuma S, Kokusho R. A Conserved Glycine Residue Is Required for Proper Functioning of a Baculovirus VP39 Protein. J. Virol. 2017;91(6). pmid:28077638.
17. Li A, Zhao H, Lai Q, Huang Z, Yuan M, Yang K. Posttranslational Modifications of Baculovirus Protamine-Like Protein P6.9 and the Significance of Its Hyperphosphorylation for Viral Very Late Gene Hyperexpression. J. Virol. 2015;89(15):7646–59. pmid:25972542.
18. Li K, Wang Y, Bai H, Wang Q, Song J, Zhou Y, et al. The Putative Pocket Protein Binding Site of Autographa californica Nucleopolyhedrovirus BV/ODV-C42 Is Required for Virus-Induced Nuclear Actin Polymerization. J. Virol. 2010;84(15):7857–68. pmid:20484515.
19. Yuan M, Wu W, Liu C, Wang Y, Hu Z, Yang K, et al. A highly conserved baculovirus gene p48 (ac103) is essential for BV production and ODV envelopment. Virology. 2008;379(1):87–96. pmid:18656219.
20. McCarthy CB, Dai X, Donly C, Theilmann DA. Autographa californica multiple nucleopolyhedrovirus ac142, a core gene that is essential for BV production and ODV envelopment. Virology. 2008;372(2):325–39. pmid:18045640.
21. Alfonso V, Maroniche GA, Reca SR, López MG, del Vas M, Taboga O. AcMNPV core gene ac109 is required for budded virion transport to the nucleus and for occlusion of viral progeny. PLoS One. 2012;7(9):e46146. pmid:23049963.
22. Wolgamot GM, Gross CH, Russell RLQ, Rohrmann GF. Immunocytochemical characterization of p24, a baculovirus capsid-associated protein. J Gen Virol. 1993;74(1):103–7. pmid:8423444.
23. Belyavskyi M, Braunagel SC, Summers MD. The structural protein ODV-EC27 of Autographa californica nucleopolyhedrovirus is a multifunctional viral cyclin. Proc. Natl. Acad. Sci. U. S. A. 1998;95(19):11205–10. pmid:9736714.
24. Vanarsdall AL, Okano K, Rohrmann GF. Characterization of the Role of Very Late Expression Factor 1 in Baculovirus Capsid Structure and DNA Processing. J. Virol. 2006;80(4):1724–33. pmid:16439529.
25. Lai Q, Wu W, Li A, Wang W, Yuan M, Yang K. The 38K-mediated Specific Dephosphorylation of the Viral Core Protein P6.9 Plays an Important Role in the Nucleocapsid Assembly of Autographa californica Multiple Nucleopolyhedrovirus. J. Virol. 2018;JVI.01989-17. pmid:29444944.
26. Guan Z, Zhong L, Li C, Wu W, Yuan M, Yang K. The Autographa californica Multiple Nucleopolyhedrovirus ac54 Gene Is Crucial for Localization of the Major Capsid Protein VP39 at the Site of Nucleocapsid Assembly. J. Virol. 2016;90(8):4115–26. pmid:26865720.
27. Yuan M, Huang Z, Wei D, Hu Z, Yang K, Pang Y. Identification of Autographa californica nucleopolyhedrovirus ac93 as a core gene and its requirement for intranuclear microvesicle formation and nuclear egress of nucleocapsids. J. Virol. 2011;85(22):11664–74. pmid:21880748.
28. de Jong J, Arif BM, Theilmann DA, Krell PJ. Autographa californica Multiple Nucleopolyhedrovirus me53 (ac140) Is a Nonessential Gene Required for Efficient Budded-Virus Production. J. Virol. 2009;83(15):7440–8. pmid:19457997.
29. Liang C, Li M, Dai X, Zhao S, Hou Y, Zhang Y, et al. Autographa californica multiple nucleopolyhedrovirus PK-1 is essential for nucleocapsid assembly. Virology. 2013;443(2):349–57. pmid:23768784.
30. Marek M, Merten O-W, Galibert L, Vlak JM, van Oers MM. Baculovirus VP80 Protein and the F-Actin Cytoskeleton Interact and Connect the Viral Replication Factory with the Nuclear Periphery. J. Virol. 2011;85(11):5350–62. pmid:21450830.
31. Li S, Wang M, Shen S, Hu Z, Wang H, Deng F. The FP25K Acts as a Negative Factor for the Infectivity of AcMNPV Budded Virus. PLoS One. 2015;10(5):e0128471. pmid:26020780.
32. Gandhi KM, Ohkawa T, Welch MD, Volkman LE. Nuclear localization of actin requires AC102 in Autographa californica multiple nucleopolyhedrovirus-infected cells. J Gen Virol. 2012;93(Pt 8):1795–803. pmid:22592260.
33. Zhang M-J, Tian C-H, Fan X-Y, Lou Y-H, Cheng R-L, Zhang C-X. Bombyx mori nucleopolyhedrovirus ORF54, a viral desmoplakin gene, is associated with the infectivity of budded virions. Arch. Virol. 2012;157(7):1241–51. pmid:22446883.
34. Biswas S, Willis LG, Fang M, Nie Y, Theilmann DA. Autographa californica Nucleopolyhedrovirus AC141 (Exon0), a Potential E3 Ubiquitin Ligase, Interacts with Viral Ubiquitin and AC66 To Facilitate Nucleocapsid Egress. J. Virol. 2018;92(3):JVI.01713-17. pmid:29142135.
35. Tomalski MD, Eldridge R, Miller LK. A baculovirus homolog of a Cu/Zn superoxide dismutase gene. Virology. 1991;184(1):149–61. pmid:1871962.
36. Mikhailov VS, Rohrmann GF. Baculovirus replication factor LEF-1 is a DNA primase. J. Virol. 2002;76(5):2287–97. pmid:11836407.
37. Feng G, Krell PJ. Autographa californica multiple nucleopolyhedrovirus DNA polymerase C terminus is required for nuclear localization and viral DNA replication. J. Virol. 2014;88(18):10918–33. pmid:25008932.
38. Yu M, Carstens EB. Identification of a domain of the baculovirus Autographa californica multiple nucleopolyhedrovirus single-strand DNA-binding protein LEF-3 essential for viral DNA replication. J. Virol. 2010;84(12):6153–62. pmid:20357098.
39. Nagamine T, Sako Y. A Role for the Anti-Viral Host Defense Mechanism in the Phylogenetic Divergence in Baculovirus Evolution. Ling E, editor. PLoS One. 2016;11(5):e0156394. pmid:27244571.
40. Stewart TM, Huijskens I, Willis LG, Theilmann DA. The Autographa californica Multiple Nucleopolyhedrovirus ie0-ie1 Gene Complex Is Essential for Wild-Type Virus Replication, but either IE0 or IE1 Can Support Virus Growth. J. Virol. 2005;79(8):4619–29. pmid:15795248.
41. Sokal N, Nie Y, Willis LG, Yamagishi J, Blissard GW, Rheault MR, et al. Defining the roles of the baculovirus regulatory proteins IE0 and IE1 in genome replication and early gene transactivation. Virology. 2014;468–470:160–71. pmid:25173193.
42. Lin G, Blissard GW. Analysis of an Autographa californica multicapsid nucleopolyhedrovirus lef-6-null virus: LEF-6 is not essential for viral replication but appears to accelerate late gene transcription. J. Virol. 2002;76(11):5503–14. pmid:11991978.
43. Okano K, Vanarsdall AL, Rohrmann GF. Characterization of a baculovirus lacking the alkaline nuclease gene. J. Virol. 2004;78(19):10650–6. pmid:15367632.
